# Fixing the ecosystem: Conservation, crisis and capital in Rwanda's Gishwati Forest

**DOI:** 10.1177/2514848619826576

**Published:** 2019-03-06

**Authors:** Nathan Clay

**Affiliations:** University of Oxford, UK

**Keywords:** Conservation, development, ecosystem approach, landscape approach, neoliberalization, socioecological fix

## Abstract

Conservation-development projects are increasingly enacted across large expanses of land where human livelihoods hang in the balance. Recent initiatives–often called ‘landscape approaches’ or ‘ecosystem-based’ conservation–aim to achieve economic development and conservation goals through managing hybrid spaces. I argue that the landscape/ecosystem approach is a socioecological fix: an effort to resolve social-environmental crises through sinking capital (financial, natural, and social) into an imagined ecosystem. Rwanda’s Gishwati Forest has been the locus of diverse crises and fixes over the past 40 years, including an industrial forestry and dairy project, a refugee settlement, a privately managed chimpanzee sanctuary, a carbon sequestration platform, and, most recently, an “integrated silvo-pastoral conservation landscape.” This paper considers how these governance schemes have intersected with broader processes of agrarian change to generate crises that subsequent conservation/development projects then attempt to resolve. I demonstrate how visions for ecosystems privilege certain forms of governance around which imagined socioecological histories are mobilized to frame problems and legitimize certain solutions, technologies, and actors. The Gishwati ecosystem and its fixes are repeatedly defined through an imaginary of crisis and degradation that engenders large-scale landscape modification while foreclosing reflection about root causes of crises or how these might be addressed. Thus, even while conservation/development paradigms have shifted over the past 40 years (from *separating* people and nature to *integrating* them in conservation landscapes), this crisis-fix metabolism has consistently generated livelihood insecurity for the tens of thousands of people living in and around Gishwati. Imagining and enacting more just and inclusive social-environmental landscapes will require making space for diverse voices to define ecosystem form and function as well as addressing deeply rooted power imbalances that are at the heart of recurrent crises.

## Introduction

In August 2014, the World Bank commenced its Landscape Approach to Forest Restoration and Conservation (LAFREC) project in the *Gishwati* region of western Rwanda. This $10 million USD scheme was intended to “improve the environment, local livelihoods, and climate resilience” in the *Gishwati* region of western Rwanda (Tumwebaze, 2014). The objective to “demonstrate landscape management for enhanced environmental services and climate resilience in one priority landscape” (World Bank, 2014: 2) is in line with other “landscape” and “ecosystem-based” approaches of environmental governance. These approaches have proliferated worldwide over the past two decades. They seek to achieve win–win outcomes in conservation and development by managing large swaths of human-dominated landscapes that have high ecological value (Reed et al., 2015; Sandker et al., 2012; Zimmerer et al., 2004). However, given LAFREC's explicit aim of *integrating* livestock grazing and reforestation makes the project an ironic bookend to a sequence of environmental management schemes in Gishwati that began in 1980 when a World Bank project emphasized *separating* livestock from cloud forests. This earlier project was rooted in a conservation paradigm which held that forest must be isolated from human uses to be protected. Thousands of hectares of “degraded” forest were razed between 1980 and 1990, with pasture established in its place in an effort to pull local communities away from grazing cattle in the remaining forest. In stark contrast, the LAFREC project has since 2014 orchestrated the planting of thousands of hectares with native tree species in effort to rehabilitate the landscape and establish a forest corridor. Perhaps speaking to the evolution of conservation–development models, the project stresses the need to integrate human uses with ecosystem services by promoting afforestation and ecosystem-based management.

LAFREC is not by any means unique. Similar ecosystem-based initiatives have emerged worldwide in terrestrial and aqueous environments across the global North and South over the past few decades (Zimmerer et al., 2004; Clay, 2016). They are widely implemented by many of the largest global conservation organizations, including the Worldwide Fund for nature, Wildlife Conservation Society, and The Nature Conservancy, in addition to the World Bank Group. These “integrated landscape approaches” or “ecosystem-based” approaches aim to avoid pitfalls of previous conservation–development schemes by facilitating inclusive stakeholder engagement in land-use planning and management across large expanses of land and water. This apparent transition away from sectoral management and towards integrated management of ecosystems would seem to indicate an exchange of modernist paradigms bent on separating the “natural” from the “human” for approaches that can better account for complex society–environment interaction. However, reliance on ecosystems as management units can serve to depoliticize environmental conservation (Cohen and Bakker, 2014) while promoting neoliberal visions of conservation through economic growth that can further marginalize human populations (Clay, 2016; Bluwstein, 2017). A recent synthesis of integrative landscape approaches points to persistent challenges that these approaches have faced due to landscape complexity and power asymmetries (Ros-Tonen et al., 2018).

With a case study of Gishwati, Rwanda, this paper illustrates how ecosystem-level environmental governance emerges through longer term metabolic processes of social-environmental change. I examine the ascendant paradigm of ecosystem-based conservation as a “spatial fix” (Harvey, 1981), wherein capital (financial, natural, and social) is sunk into the landscape in effort to hold social and environmental crises at bay. To argue that the current trend of ecosystem-based conservation is a spatial fix that binds conservation and development agendas, I build on a raft of recent literature on “ecological,” “environmental,” “eco-scalar,” and “socioecological” fixes (Bakker, 2004; Castree, 2008; Cohen and Bakker, 2014; Ekers, 2015; Ekers and Prudham, 2017; McCarthy, 2015; Swyngedouw, 2013). While this literature has largely concentrated on urban contexts in the global North, I demonstrate that the socioecological fix concept helps us understand how ecosystems emerge as material-discursive spaces through attempts to install neoliberal natural resource management protocols in the global South.

To this end, this paper documents how environmental governance schemes over the past 40 years in Rwanda's Gishwati forest region have responded to socioecological crises in ways that (1) purportedly offset negative environmental effects (including forest degradation, climate change, and biodiversity loss) that emerge at various scales and (2) simultaneously attempt to shore up the means of production for economic growth. I narrate how 1980s paradigms emphasizing separate productive uses (e.g. spatially segregating forestry from livestock grazing and converting forest to pasture in effort to enhance overall economic viability of the landscape) have morphed into a contemporary vision of an “integrated silvo-pastoral landscape” (World Bank, 2014) in which forest restoration and management activities are organized at the ecosystem level. In the decades between, Gishwati was managed as a refugee settlement, a private forest for chimpanzee research and conservation, and a UN flood disaster zone and reforestation/carbon sequestration platform. I show that each conservation and development scheme is at once shaped by past crises and generative of future crises that conservation/development actors implement socioecological fixes to resolve. These governance schemes are implemented in ways that uphold as well as redefine existing power relations and social inequalities.

Environmental governance schemes that are intended to manage landscapes in integrated ways can be considered hybrids themselves, for they continuously wrestle with conflicting notions of society–environment interaction (Zimmerer, 2000; Clay, 2016). As paragons of re-scaled environmental governance (Cohen and McCarthy, 2015), there is need to critically assess how “landscape-based” and “ecosystem-based” approaches to conservation and development rework society–environment relations through their privileging of ecological scales (Forsyth, 2005; Gruby and Basurto, 2013). This paper considers the claims of integrative multi-stakeholder management that supposedly set landscape approaches apart (Sayer et al., 2014) from earlier schemes of environmental governance and development that were based on more overtly binary models of society–environment interaction. While landscape approaches attempt to address earlier frustrations over separating nature and society and aim to bend colonial articulations of conservation and development toward more inclusive futures, I argue that in practice they continue to operate with logics of modernization, techno-scientific expertise, and exclusion. With a case study of western Rwanda, I show that ecosystem-based conservation manifests through the erasure of insalubrious histories of conservation and development injustices. This blurs the social-environmental struggles that have shaped the landscape through cycles of uneven development and thereby facilitates the illusion of a fresh start for landscape governance initiatives to flourish. I point to the practical value of assessing how such landscapes have been shaped by diverse, evolving, and often conflicting views of what constitutes environment–society interaction.To envision more just and inclusive governance, we must first identify how these contested socionatural histories are imprinted on the landscape and seek to correct processes that have continuously led to uneven outcomes for humans and non-humans.

## Governing nature–society hybrids

Biodiversity conservation initiatives have increasingly targeted human-dominated landscapes (Zimmerer et al., 2004), often through governance regimes that link states, civil society, and non-governmental organizations (NGOs) (Lemos and Agrawal, 2006). Research on conservation in the Anthropocene must account for this explosion of nature–society hybrids and global assemblages of environmental governance (Lorimer, 2012; Ogden et al., 2013; Robbins and Moore, 2013; Zimmerer, 2000).

Burgeoning scholarship in political ecology and allied fields contributes to such understanding with recent work illustrating how biophysical processes in continuous states of transformation intersect with cultural ideas and narratives to *co-produce* socio-natural hybrids (Bakker and Bridge, 2006; Goldman, 2009; Robbins, 2012). The co-production analytic helps see environmental governance as a nuanced process rather than a totalizing force (Bakker, 2009) and can generate empirical detail about how governance schemes simultaneously rework landscapes and react to material social-environmental aspects of those landscapes (Robbins, 2001). In these co-constitutions of nature and society, power and agency are fluid, composed through dynamic relationships among diverse social actors (Himley, 2009) and non-humans (Swyngedouw, 2003). In this work, paradigms of environmental governance are understood as fusions of biophysical processes and social imaginaries that are tied to political economic agendas and mobilized through institutional practices (Swyngedouw, 2013). As Bridge and Perreault (2009: 485) put it, this environmental governance scholarship generates “nuanced analyses of how power is produced and exercised over and through the non-human world.”

### “Ecosystem” as integrated conservation–development space

Political ecologists have written on a wide range of conservation and development forms. With case studies in sub-Saharan Africa, this work has demonstrated how boundary-making in national parks and surrounding areas can follow colonial patterns of control by criminalizing and policing territory, resources, and activities to shift local resource use into the purview of external management (Goldman, 2003; Neumann, 1998). Even where conservation and development programs aim to include participation of resource users through community-based approaches, decision-making can remain the realm of external “experts” through reliance on techno-scientific procedures of knowledge generation and management (Goldman, 2009; King, 2009).

Recent environmental governance schemes aim to address past failures of separating nature and society by more effectively accounting for multiple land uses—human and non-human—within conservation spaces. Efforts to blend biodiversity conservation with activities to generate economic growth have greatly expanded in recent years (Arts et al., 2017; Zimmerer et al., 2004). Often under the umbrella of “landscape” or “ecosystem-based” conservation, these schemes attempt to integrate diverse actors who use resources in a given area (typically including the state, private sector, and local communities) into environmental management efforts (Lemos and Agrawal, 2006; Reed et al., 2015). This means that territories of environmental conservation now commonly extend beyond state-run protected areas to encompass diverse spaces essential to human livelihoods (Clark et al., 2009; Miller and Hobbs, 2002). Indeed, a core aim of ecosystem/landscape approaches to conservation is to cooperatively manage a range of land uses across substantially larger spatial extents than were previously afforded by protected area systems. The need for larger conservation territories is upheld by ecological research that suggests the importance of maintaining breeding populations of large-bodied mammals, which often require expanses of contiguous habitat (Sayer, 2009). As such, environmental governance is re-scaled, a process that creates the ecosystem as a new governance object (Cohen and McCarthy, 2015). A second objective of ecosystem approaches is to install platforms of multi-stakeholder management that may better account for livelihoods of multiple groups living in these enlarged conservation territories (Arts et al., 2017).

One strand of scholarship on ecosystem-based conservation has focused on assessing the policy environment and identifying strategies for enhancing stakeholder collaboration, which many consider a precursor to achieving synergy between biodiversity conservation goals and human wellbeing (Arts et al., 2017; Boedhihartono et al., 2015; Reed et al., 2015; Sayer et al., 2014; van Oosten et al., 2014). For example, a recent study of Rwanda's Gishwati region aimed to develop strategies to overcome “conflicting policy objectives” surrounding landscape-level conservation through multi-stakeholder decision making (van Oosten et al., 2018: 68). However, others find that such strategies of stakeholder engagement may be ineffective due to power imbalances among the diverse actors involved in ecosystem-based conservation, which can include indigenous groups, conservation NGOs, and transnational resource extraction companies (Clay, 2016). Given the presence of powerful actors, Forsyth (2005) cautions that ecosystem-based conservation can encourage discourse coalitions, wherein elite groups of managers select which narratives about past resource use to draw from so as to legitimize their preferred environment–society interactions. Indeed, the logics and protocols of ecosystem-based conservation appear strikingly consistent with other spatial forms of conservation in that they privilege expert knowledges to delimit territories for management (Adams et al., 2014; Gruby and Basurto, 2013; Sievanen et al., 2013). Ecosystem-based conservation has been demonstrated to intersect with broader political processes related to indigenous territory claims (Gruby et al., 2017) and to constrain resource access and marginalize certain livelihoods due to deeply rooted social inequalities (Clay, 2016). The present paper builds on this work by considering how ecosystem approaches to management emerge through the convergence of diverse paradigms of human–environment interaction, where the ecosystem becomes a site to fix socioecological crises.

### Ecosystem as site of socioecological fix

Recent work has extended Harvey's (1981) notion of spatial fixes (mitigating crises by investing in built landscapes to renew processes of accumulation) to consider how capital is sunk into biophysical landscapes to alleviate social and ecological crises. Ekers and Prudham (2015) term this a *socioecological fix*. I suggest that ecosystems can become sites of socioecological fix, emerging through responses to crises of capitalist development and environmental sustainability. Socioecological fixes address social-environmental crises of capitalist production systems, including crises surrounding the legitimacy of organizations (Ekers and Prudham, 2018). As Cohen and Bakker (2014: 133) explain: “these fixes are often undertaken to address crises brought about by patterns of capital accumulation and uneven development, such as environmental degradation, changes in labor markets, and an unwillingness by actors to absorb costs of producing negative environmental externalities.” For example, with a case study of forestry landscapes in 1930s Canada, Ekers (2015) demonstrates that investments in forest stations were intended to address both depression-era unemployment and the legitimacy of state forestry operations while simultaneously securing opportunities for accumulation. Socioecological fixes are enacted through shifts in social regulatory processes and entail the production of space and nature (Ekers and Prudham, 2015), often transforming relationships among humans and non-humans (McCarthy, 2015), including targeting human bodies as the sites of spatial fix (Collard and Dempsey, 2013; Guthman, 2015). While socioecological fixes are enacted in effort to address challenges of uneven development, they can in practice serve to reproduce the status quo. For example, as McCarthy (2015) argues about the socioecological fix of transitioning to renewable energy, greener production could engender transitions of simple substitution that do little to transform systemic issues that have long produced inequalities.

The present paper builds on this scholarship by exploring how ecosystem-based conservation—despite its appearance of resolving numerous social and environmental crises—can similarly enable the persistence of uneven processes of development (cf. Smith, 1984). This paper makes three key contributions to the literature on socioecological fixes. First, it addresses a central unanswered question concerning how socioecological fixes play out over longer time periods, including whether they exacerbate crises (Ekers and Prudham, 2015; Moore, 2011). With a case study of overlapping environmental governance schemes in Gishwati over a 40-year period, I demonstrate how socioecological crises are co-constitutive with the sequence of socioecological fixes. Secondly, I respond to calls for work on socioecological fixes to engage with socioecological change and the political nature of this change in terms of landscape metabolism, or “the conjoined social and ecological character of historical–geographical processes of fixed capital formation” (Ekers and Prudham, 2017: 1373). Specifically, I elaborate the processes by which capital is fixed in the “ecosystem” through the involvement of the state, civil society, and NGOs. This connects with recent work that demonstrates how conservation continues to be embedded in neoliberal development processes (Brockington and Duffy, 2011). Third, while scholarship on socioecological fixes has drawn almost exclusively on empirical material from the global North, I illustrate the applicability of the concept to economic development and conservation interventions in the South. I show that the Gishwati landscape has been produced through a series of crises and attempts to address them, highlighting how narratives and practices of conservation and development converge to generate synergistic legitimacy to surmount these ongoing crises.

## Fixing the Gishwati ecosystem

The following analytical narrative draws from assessment of historical and contemporary project and policy documents and related material as well as from key informant interviews conducted during two research visits to Gishwati in 2012 and 2014. The Gishwati landscape is situated in western Rwanda, the ghost of a former cloud forest purported to have once covered around 110,000 hectares (Plumptre et al., 2001). Averaging 2500 meters above sea level across a series of steep peaks and narrow valleys, Gishwati stretches north to south along the Congo–Nile crest, a mountainous ridge that separates rivers flowing east into the Nile watershed from those draining west into the Congo River basin. The Gishwati landscape currently consists of about 800 hectares of cloud forest surrounded by agro-pastoral areas and tea estates. Ecologically speaking, the forest is situated within the wider Albertine Rift tropical montane forest ecosystem, a string of cloud forests that may have once been contiguous across the Congo–Nile crest. Some suggest that Gishwati served as a bridge linking cloud forests to the north (now Virunga/Volcanoes National Park) and to the south (now Nyungwe National Park) (Plumptre et al., 2001). This historical imaginary of the forest continues to structure environmental governance and development aspirations in the region.

Gishwati's socionatural history is in many ways a microcosm of the arc of society–environment interaction in Rwanda. Beginning centuries ago, the area was home to forest-dwelling hunter-gatherer people known as *Batwa*. Gishwati's forests began to be cleared in the 19th century by agriculturalist *Bahutu* and pastoralist *Batutsi* groups (Newbury, 1988). The political system in pre-colonial Rwanda was hierarchical, structured around these ethnic identities, which were closely tied to livelihood and land use (Newbury and Newbury, 2000). Agriculturalist Bahutu majority were presided over by pastoralist Batutsi. These identities were fluid in that individuals could become Tutsi if they accumulated enough cattle, suggesting that class differences had ethnicity mapped onto them, rather than ethnicity driving class differentiation (Dorsey, 1983). A historically marginalized people, Batwa were incorporated into expanding agro-pastoralist societies which divested them of traditional forest use rights and compelled them to adopt alternative livelihoods (Lewis, 2000). These hierarches were maintained through Rwanda's colonial period (1987–1962), and often used by the state and development organizations in ways that overlook more pervasive and complex issues of social inequality (Daley, 2006). Gishwati was incorporated as a national forest area in the 1930s, yet deforestation continued throughout the 20th century. As in the rest of Rwanda, subsistence agriculture was the predominant livelihood in Gishwati through the 1980s, although state investments in commercial tea production in the 1960s enabled some interaction with international markets. Tea commercialization also led to further deforestation, with cloud forest cleared to plant tea and to supply furnaces drying tea leaves (von Braun et al., 1991). In the 1980s, international development operations directed funds into large-scale measures to enhance agricultural productivity and soil conservation throughout Rwanda.

### Modernizing livestock and forestry systems to save the forest (1980–1994)

The first of several environmental governance interventions in Gishwati, the World Bank's Integrated Forestry and Livestock Development (GBK by acronym) projects emerged in 1980 from this context of commercialization, deforestation, and marginalization. By this time, Gishwati Forest covered around 28,000 hectares, with only 5000–6000 hectares of that identified as old-growth primary forest and the rest considered degraded (World Bank, 1980). The first iteration of GBK (a 23.6 million USD project) ran from 1980 to 1987. The second iteration (14.1 million USD) was active from 1988 to 1994. Underlying these schemes was a modernizing ethos that painted the Gishwati region as degraded and undeveloped to manufacture the need for techno-scientific development intervention. The projects were implemented across an area of about 110,000 hectares that comprised the former Gishwati Forest. In effort to simultaneously protect biodiversity and contribute to regional economic growth, the GBK projects centered on clearing forest patches identified as “degraded,” which were then converted to “improved pastures” and forestry plantations of pine species. The dual objectives were to achieve economies of scale in dairy and forestry and to prevent small-scale grazing of cattle in forest parcels, which was identified by the Bank as the principal cause of forest degradation. Justifying this ambitious program of landscape engineering was a narrative that positioned ecological modernization as the solution to a land degradation crisis that was seen to be caused by inappropriate subsistence land use practices. As the Project Completion Report puts it, the project rested

… on the reasonable argument that in order to conserve the Gishwati natural forest, it would be necessary to get the native cattle out of the forest, and in order to make it attractive for cattle owners to do so, alternative pasture areas would have to be established. (World Bank, 1991: iii)

This “technical assistance” mandate was premised on the idea that the only way to stop the informal grazing that was said to be destroying the forest was to separate nature and society by establishing separate spaces for commercial dairy and commercial plantation forestry—in short: commodifying the landscape to save it.

This approach fit with dominant conservation and development models in Rwanda and elsewhere in the 1980s. As the most densely populated country in Africa, population pressure was continuously identified as a threat in Rwanda, and the government had long sought intensification of resource-based activities to avert economic and environmental catastrophe (World Bank, 1980). GBK's strategy of intensification also aligned with the protectionist model of conservation widely practiced in the 1980s, which encouraged segregation of areas for conservation (e.g. the remaining, undegraded Gishwati forest) from areas for commercial agriculture. GBK was initially proposed to enhance Rwanda's capacity for industrial forestry, with the dairy commercialization component added during negotiation (World Bank, 1980). These projects, which were even then seen as controversial (Ford, 1990), reworked the socionatural landscape of Gishwati. The GBK projects hewed to an economic optimization mindset that was also seen as necessary to simultaneously protect the environment. As the initial project outline puts it, the objective was to “make optimal use of the high, but presently underutilized, agricultural potential of the Gishwati area while carefully protecting the ecological balance” (World Bank, 1980: 22). At the same time, Gishwati was identified as the only suitable area for large-scale plantations because of its higher soil fertility, which is necessary for production of saw-timber. Paradoxically, Gishwati was thus selected both to protect its ecological fragility and because of its ecological predisposition for plantation forestry.

These contradictory narratives underwrote the unfolding of GBK as a socioecological fix. Further contradictions emerged in the project's design and implementation. The dairy commercialization component was added midway through planning due to recognition of pre-existing subsistence livestock production in Gishwati (there were approximately 26,000 cattle in the area in 1980), which the Bank worried could be lost if it emphasized only forestry. The Government of Rwanda also strongly desired the dairy commercialization component. While project documents disclose that there was disagreement about including a dairy component, dairy commercialization also become fundamental to the overall project, which assumed the name “Integrated Livestock and Forestry Development”. The decision to “integrate” livestock and forestry commercialization was ultimately rationalized as essential to save the remaining forest from destruction by subsistence cattle grazing (World Bank, 1980). As subsequent reports indicate, the livestock component “overwhelmed all the others” (World Bank, 1991: 4). The contradictory way in which GBK was designed and implemented exposes how socioecological fixes emerge through negotiations among multiple actors about the active substantiation of regulatory processes that hinge on the reworking of socionature. In Gishwati, transformation of socionature was intensive and extensive. The final Project Completion Report described the premise and goals of GBK as follows:

Recognizing the environmental and economic threat posed by rapid destruction of natural forests, both projects were aimed at: (i) expanding the source of wood products through industrial plantation and rural afforestation; (ii) enhancing the value of protected natural forest ecosystems; (iii) developing a high productivity cattle industry from degraded natural forests in the Gishwati area; and (iv) strengthening the institutional capacity of the forest and livestock agencies involved. In the Gishwati area, it was anticipated that most traditional nomadic cattle owners in the forest reserve would adopt an intensive dairy farming technique. (World Bank, 1996)

These summarized objectives capture how the GBK projects sought a socioecological fix in response to a perceived crisis of forest degradation that was pinned as both economic and environmental. This fix was also highly technical in that it required (1) identifying and removing degraded forest, (2) establishing an industrial plantation of pine trees for timber production, (3) establishing pasture where forest previously was and intensifying farming systems outside of the forest boundaries by planting forages; (4) facilitating the adoption of “improved” cattle breeds (Swiss Brown cattle were imported from Europe). Further capital was sunk into the construction of a milk processing center and a sawmill. Together, these capital-intensive and labor-intensive ventures were to further Rwanda's modernization—a goal of both the Bank and the Government of Rwanda—by creating a landscape of combined industrial forestry and “high productivity cattle industry” while contributing to environmental security (World Bank, 1980).

**Figure 1.**
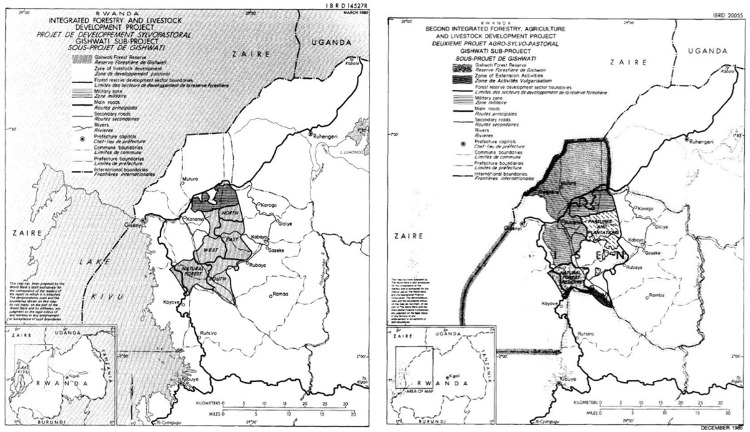
Maps of boundaries for the World Bank's Livestock and Forestry Development projects and Gishwati forest reserve; from project appraisal document (World Bank, 1980, 1996).

GBK was implemented through the establishment of a “long-term land use plan,” maps of which are displayed in Figure 1. This platform of environmental governance initially separated Gishwati into 5000 hectares of remaining primary forest, 10,000 hectares of pine plantation, at least 5000 hectares of pasture, and 5000 hectares for a military zone. It was anticipated that about 4000 hectares of “degraded forest” would be cut down and divided equally between pasture and pine plantation. Forestry professionals and agricultural extension agents were charged with implementing these various project components, which included selecting areas for either pasture or forestry and ensuring that nearby agricultural communities intensified farming systems by incorporating production of fodder grasses to offset the seasonality of grazing in Gishwati. In this way, the socioecological fix reworked subsistence production systems well beyond the forest reserve's boundaries. Following Bakker (2009), the overall premise of GBK here appears to have been to internalize the negative socioecological externalities emanating from unruly subsistence livelihoods and unpredictable biophysical environments by transitioning to management via industrial operations that are clearly demarcated spatially.

This socioecological fix was also highly political in that it required substantial altering of regulatory systems. Adjusting the political economy of livestock management was a goal of the project, which recognized the historical legacy (rooted in precolonial hierarchical systems) of management “whereby a farmer raised the cattle of a traditional chief is still commonly practiced” (World Bank, 1980: 9). Onto this system, GBK aimed to superimpose a distinctly neoliberal governance regime. While farmers were accustomed to the government distributing seedlings free of charge, costs for rural woodlots were to be covered by charging farmers for each seedling they purchased to plant on their land (World Bank, 1980). Farmers were expected to “contribute to cost recovery” through payments for pharmaceuticals and veterinary treatments at newly established animal health centers. This focus on individual farmers was also considered a serious risk, as a GBK project document notes: “the main risk of Gishwati livestock component is the time required for farmers to adopt and manage efficiently the proposed technical package, both in the farming zone outside the forest, and in the grazing zone in the forest” (World Bank, 1980: 49).

In 1991, a Project Completion Report declared the first phase of GBK a failure in terms of environmental and economic objectives. Environmentally, the project was considered unsuccessful in that “uncontrolled livestock and human activity” continued (World Bank, 1991). Reasons cited for failure to meet economic potential included high cost of pasture establishment, overabundance of local cattle breeds (that is, low rate of adopting European breeds) that could not biologically produce more milk even with improved pasture) and capturing of rents from the dairy project by external elites who took advantage of artificially high milk prices. In contrast, forestry components were considered successful “because they re-enforced the government's ongoing program and commitment” (World Bank, 1991: iv), suggesting how socioecological fixes can uphold established models of state–society relation. The pasture establishment component of GBK, on the other hand, was noted as controversial and flawed in its insufficient preparation with regard to social dynamics despite the excellence and abundance of biophysical and technological prerequisites:

Excellent climatic conditions existed for establishing very productive high altitude pastures, and the technology had been proven in neighboring Zaire; had this potential been correctly estimated, it would have been also clear that low productivity native cattle would be unable to capitalize on this potential, and grade cattle would be needed. Also, had the social affinities in the region as well as the consequences of an artificially high milk price been better understood, it could have been foreseen that private interests from the outside would have come in. (World Bank, 1991: iii)

This passage illustrates how society and environment are entangled within this socioecological fix of landscape-level management in Gishwati. It also illustrates how this fix generates additional socioecological crises. While the initial plan was to require individuals wishing to use pasture privately to contribute their own labor to establishing pasture, this was deemed inefficient and pasture was cleared by hired labor using project funds. As a result, absentee livestock owners took control of large swaths of pasture. Establishment of pasture made the pre-existing local cattle breeds and management system obsolete, leading to a crisis of underproduction for farmers living nearby (see O'Connor, 1988). At the same time, hybrid cattle (progeny of local cattle and several imported Swiss brown bulls) were distributed to wealthy (and often absentee) private cattle owners and milk production among hybrid cows exceeded initial project estimates by a factor of four. Elites captured a huge portion of these benefits. As the Completion Report notes, “51% of the total [pasture] have been allocated in unit areas of 30 ha or more to small groups of less than 10 beneficiaries very often belonging to the same family and including government officials and project staff” (World Bank, 1991: 7). While the project was set up to stop farmers from grazing in the forest, it was found that those farmers did not enroll in contracts to use newly established pasture due to fear of paying a cattle tax and disinclination to adopt intensive practices due to the higher risk entailed (World Bank, 1991). This shows how uneven geographies of development can emerge through socioecological fixes, within which power differentials are deeply entrenched. Further illustrating this point, a Project Completion Report acknowledges that the skewed distribution of benefits was essential to enumeration of the project's quantitative successes and the speed of achieving these targets:

the involvement of influential and better-off people has also had a few positive aspects. It must be candidly admitted that without their participation, the high potential of the Gishwati area for the dairy industry would have been much slowly exploited. For instance all the ten livestock owners who recorded more than 70 1/pasture of 30 ha/day in 1987 were all among this class of beneficiaries. Also it has certainly been stimulating for the P.M.U. staff to have as clients high officials who had a say in their promotion and reassignment” …. “the underestimation of the potential of the livestock enterprises led to overlook the pressure the pastures would trigger from private interests in a country like Rwanda where such highlands are among the last opportunities to acquire new lands. (World Bank, 1991: 8)

The above passages exemplify the tendency of development institutions to write off socionatural complexities and depoliticize the uneven outcomes of socionatural transformation, instead depicting project failures as arising from incorrect estimates or accounting (Ferguson, 1990). These passages also show, more insidiously, that development project “successes” can at times rely on the further entrenchment of social inequality in the socionatural landscape. By the time this first Project Completion Report was published (in 1991), the second phase of GBK was already well underway (since 1987) in Gishwati. Further illustrating the tendency of development institutions to evade society–environment complexities that they help produce, the project's second iteration aimed to correct earlier mistakes by further embedding a neoliberal ethos. Even more control was allotted to the private sector and individuals, who were expected to take on management responsibilities, and the project aimed to increase the number of households enrolled in dairy commercialization from 40,000 to 75,000. Reacting to accusations that the Bank was complicit in forest destruction, the project's second phase also diverted attention to building capacity for conservation management and research in Gishwati and Nyungwe national forests. These moves towards local level management reflect broader patterns of decentralization that were being rolled out in forestry and conservation landscapes throughout the developing world in the 1990s alongside other mechanisms of neoliberal environmental governance (McCarthy and Prudham, 2004; Ribot et al., 2006). In Gishwati, the Bank appears to have doubled down on these mechanisms in response to the socioecological crisis created with the first socioecological fix. In the second phase, allegedly “much more attention has been paid to cost recovery issues, milk pricing and marketing policies and land tenure problems” (World Bank, 1991: iv).

Perhaps unsurprisingly, the narrow (and segregated) economic and ecological focus of these adjustments offered little in the way of addressing uncontrolled involvement of external elites and exclusion of local producers. Any successes reported about the project's first phase turned to failures by the end of the second phase. Towards the end of the 1980s, land conflicts erupted between local and external groups surrounding both forestry and livestock zones. Struggles escalated to the point that the World Bank withdrew support for the project's livestock component in the early 1990s (World Bank, 1996). Conflicts also erupted surrounding the buffer zones set up along Gishwati and Nyungwe park boundaries. As the Project Completion Report states, “local people resented having their land expropriated and despite being employed in project activities they destroyed the buffer zones in retribution” (World Bank, 1996). Even more alarmingly, the conversion of cloud forest to pasture and pine plantations displaced several thousand Batwa, who became internally displaced in Rwanda, as there was no resettlement plan in GBK (World Bank, 1996). The last Batwa were evicted from Gishwati in the 1980s (Lewis, 2000). Displaced Batwa generally moved to Rwanda's capital, Kigali, where they led a marginalized existence (Lewis, 2006).

The above demonstrates the magnitude of socioecological transformations as the World Bank and Government of Rwanda (GOR) sunk capital into the Gishwati landscape to promote biodiversity conservation and economic growth. These transformations both relied on and exacerbated pre-existing patterns of uneven development. I next discuss how components of Gishwati's socionature persist and were reworked in the 15 years after GBK amid Rwanda's recovery from brutal civil war and genocide.

### War, refugees, and resettlement (1994–2007)

Conflicts flared in Northwest Rwanda from 1990 to 1993. The Rwandan Patriotic Front (RPF, an army composed mainly of Tutsi who were exiled following civil conflict in 1959) crossed the border from Uganda in an initial push to regain citizenship and land rights. Parallel to these incursions were diplomatic discussions between the RPF and Rwanda's then president, Juvenal Habyarimana, surrounding the re-integration of the Tutsi refugees. The GBK project came to a standstill in 1993, as the government's financial situation deteriorated and civil allegiance to the government weakened, with farmers burning government-owned plantations in statements of disapproval (World Bank, 1996). By this time, the military zone in Gishwati had become a training ground for a paramilitary militia known as *Interahamwe*. In August 1993, pressured by the World Bank and other actors who refused needed assistance to now-bankrupt Rwanda, Habyarimana signed the Arusha Accords, a treaty permitting the return of Tutsi refugees and power sharing in government and military. Following high-level meetings in April 1994 on how to implement the Accords, Habyarimana's plane was shot down, an action that served as pretext for and immediately ignited premeditated genocide against the minority Tutsi, who accounted for approximately 10% of the population in 1994. A concurrent civil war raged as the RPF advanced southward to Kigali. In the ensuing 100 days between April and July, nearly one million Rwandans were killed. A further two million fled the country and hundreds of thousands were displaced internally (UN, 1999). Rwanda's genocide was a story of a government mobilizing state resources through administrative structures to turn citizens into killers in effort to hold onto power by extinguishing an ethnic minority. It was also a story of abandonment by global powers—Belgium, France, the United States, and the UN—who failed to heed signs of impending disaster, refused to acknowledge the genocide, and shirked from military action that could have averted catastrophic loss of life (Desforges, 1999). And it is a story that has circulated in problematic ways, with complex social realities in the Great Lakes region commonly elided in favor of emphasis on ethnic difference, which states have used to manufacture continued tension (Daley, 2006).

These geopolitical elements converged in Gishwati. Concerning GBK, many of the investments made in the two phases of the project were reported destroyed and all the cattle slaughtered or taken out of the country (World Bank, 1996). During and after the war and genocide, the forest became a place of sanctuary and of fear. Tutsi took refuge in Gishwati forest during the war and post-war due to weekly raids of a nearby refugee camp by *Interahamwe* militia (USCR, 1995). In 1997, *Interahamwe* settled in the forest to hide among the trees, which incentivized further cutting of forest for safety concerns (Hanes, 2006). Instability and military activity continued in Gishwati until at least 1998, when conflict re-emerged between *Interahamwe* and RPF (UN, 1999). As the RPF took power in July 1994, around 800 thousand refugees who had fled 1959 returned. In 1996 and 1997, around 1.4 million of those who left Rwanda returned. Some of the 1959 refugees were resettled in and around Gishwati, the government having declared a small portion of the forest open for settlement (Hanes, 2006).

These political ecological complexities surrounding the Gishwati region in post-conflict Rwanda are commonly elided for the simpler maneuver of labeling refugees forest destroyers. This notion has been powerful, circulating widely in countless scientific and project reports over the past 15 years. These reports narrate a Malthusian population–environment relationship with a distinct post-conflict hue: Gishwati is portrayed as a victim of refugee resettlement and, by association, a casualty of subsistence production. Conservationists linked rapid deforestation in Gishwati to refugees (Kanyamibwa, 1998; Plumptre et al., 2001). A report on environmental threats in Rwanda claims that “The GOR's need to permanently resettle the millions of returnees since the 1994 genocide, and to provide people with fuel, agricultural land, and shelter, has led to the almost total destruction of Gishwati and Mukura forest reserves” (Chemonics, 2008: 4). In 2009, the US National Aeronautics and Space Association (NASA) released parallel satellite images of the Gishwati Forest area from 1986 and 2001, explaining that:

in the 15 years that elapsed between these images—a time that spanned the country's tragic genocide—wave after wave of refugees arrived in Gishwati Forest and began clearing it, often for subsistence farming. By 2001, only a small circular patch of native forest remained—1,500 acres of the forest's original 250,000. (http://visibleearth.nasa.gov/view.php?id=38644)

Problematically, this analysis appears to attribute to refugees all the destruction of Gishwati forest that occurred since before the 1930s. The NASA assessment was taken up by conservation-oriented websites including the Mother Nature Network, which posted the following on 11 June 2009:

NASA has released satellite images of the Gishwati Forest in Rwanda that reveal stunning destruction between 1986 and 2001. Refugees who have made their homes in the Gishwati Forest after the 1994 genocide have cleared and cultivated it for survival, leaving only 600 of the forest's original 100,000 hectares, a loss of 99.4%. (Rogers, 2009)

This illustrates how a crisis of refugees destroying the forest emerged in the wake of the GBK project. This catastrophic forest loss in Gishwati is commonly cited in Rwandan policies aiming to intensify agricultural activities while re-constituting protected areas and forest cover. For instance, the 2005 Organic Land Law draws on this narrative. The idea is also recycled in reports by international actors. For example, a UN report on sustainable development in post-conflict Rwanda notes: “Gishwati Forest, in particular, was most affected [by the war]. Initially covering an area of 23,000 ha in 1980, Gishwati shrunk to a mere 600 ha by 2002” (UNEP, 2011: 82). Depicting Gishwati as a landscape ravaged by the disorder of war, these reports pitch Gishwati as in need of orderly, modern management. However spurious the claims of the NASA analysis are, this powerful image of war-caused deforestation has travelled far and wide situating the imaginary of the region as a once-pristine landscape that was ravaged by war. For example, the site description for an agroforestry research project in the Gishwati area references the NASA analysis:

Gishwati Forest used to be one piece in a complex system of rainforests through the middle of Africa. It used to extend west beyond Lake Kivu connecting with the rainforests of the Congo, and south connecting with Nyungwe Forest. These forest systems have become fragmented due to population increase and deforestation. During the Rwandan Genocide, wave after wave of refugees arrived in Gishwati Forest and began clearing it, often for subsistence farming. By 2001, only a small circular patch of native forest remained, 1,500 acres (6.1 km^2^) of the forest's original 250,000 (NASA Earth Observatory). (Gyau et al., 2014: 5)

The wide uptake and use of these figures demonstrates the effortlessness with which misleading statistical and geospatial assessments of landcover change can travel. In the post-conflict context, such ideas have special potency, or what Anna Tsing (2005) might call “friction”, in their ability to reframe debate and mobilize resources for conservation and development agendas. In Gishwati, simplified narratives of aggregate population pressure on the resource base belie the historical political ecological complexities that shaped Gishwati. As discussed above, the GBK projects had already substantially altered the landscape, removing thousands of hectares of forest. Even before these projects began, only around 5000 hectares of primary forest remained in Gishwati. Recent analysis of remotely sensed imagery indicates that forest loss occurred in areas established by the GBK project as plantation forests (Ordway, 2015). After the war, it is unclear how much of this remaining forest was cut to prevent militia groups from hiding there. Moreover, reports indicate that resettlement of refugees was hasty and poorly managed. For instance, much of the monitoring was done by military which was disturbing for people still traumatized by recent war. After the UN authorized repatriation of refugees, there was pressure to move swiftly in order to gain favor with international donors. At the same time, international donors delayed in honoring financial commitments (Drumtra, 1995). The political economy of the way in which the refugee situation was handled thus had as much or more effect on deforestation as aggregate population pressure on forests. Nevertheless, the image of a once-grand primordial African rainforest forest decimated by refugees for subsistence farming is now nearly ubiquitous in grant proposals and project descriptions on the Gishwati region. As I demonstrate in the following sections, subsequent socioecological fixes strategically deploy this crisis narrative to generate support for landscape transformations and regulatory changes.

### Landslides, reforestation, and the ‘Forest of Hope’ (2007–2014)

A devastating material consequence of the GBK socioecological fix emerged in the mid-2000s in the form of floods and deadly landslides. By this time, the Gishwati landscape contained numerous town centers and peri-urban areas, the result of refugee resettlement patterns that had mapped onto infrastructure and settlements linked to the influx of capital from the GBK projects. The settlements swelled after 2005 as the Rwandan government evicted refugees who had settled nearer to Gishwati amid growing environmental concerns. Many of these settlements were juxtaposed dangerously close to hills that had been denuded for GBK's pasture and pine plantations. In September 2007, heavy rains caused landslides in Nyabihu district (located on the northeast edge of Gishwati, where GBK pasture and forest plantation activity had been heavily concentrated) and Rubavu district (on the northwest edge alongside the area formerly delimited as military zone). The landslides swept away people and property, killing dozens, displacing several thousand, and leaving 432 families homeless (Tumwebaze, 2007). Subsequent assessments of the landslides' causes pointed to forest destruction for subsistence agriculture, citing the misleading NASA analysis (discussed above):

“This area is not supposed to have floods, but because of the deforestation of the natural reserve of Gishwati, which is near the upstream of Rubavu town, and the intensive agriculture due to human pressure, we have periodic floods”…. “NASA notes that Gishwati's destruction is largely a result of subsistence harvesting and cultivation in the aftermath of the 1994 Genocide against Tutsi and that, overall, only 600 hectares of Gishwati's original 100,000 hectares remain.” (Ntambara, 2009)

The landslide disaster was a socionatural product, the result of years of intersecting political economic and ecological processes within which the GBK socioecological fix was entangled. Yet, the crisis was pinned on subsistence producers. Responses to the landslides emphasized evicting further families (Tumwebaze, 2009) and making maps to plan Gishwati's rehabilitation (Ntambara, 2009). These efforts by the newly established Rwanda Environmental Management Authority (REMA) coalesced into a partnership with the UN Development Program on a four-year project, entitled Reducing Vulnerability to Climate Change by Establishing Early Warning and Disaster Preparedness Systems and Support for Integrated Watershed Management in Flood Prone Areas (a 3.3 million USD project in operation from 2010 to 2014). As the project's name suggests, the 2007 floods were attributed to climate change and the project proposed a technical fix (integrated watershed management) for the rehabilitation of the Gishwati *ecosystem*:

“The project aims to reduce the vulnerability of the Gishwati ecosystems and its associated Nile-Congo crest watersheds, and the people that derive their livelihoods from it, to increased floods and droughts due to climate change. The proposed project intervention area includes regions within the crest area of Nile-Congo basins, also categorized as the Gishwati ecosystem, identified through the NAPA process as being among the most vulnerable to climate change.” …. “The project will promote and engage in ecosystem rehabilitation as a critical part of the management of disaster risk, as well as the development of risk maps, land use and settlement plans, and the application of adaptive measures, which will achieve increased ecosystem resilience to climatic shocks.” (UNDP, 2011: 2)

This language brings in climate change and reconstitutes Gishwati as an ecological landscape in need of increased resilience. Subsistence production and refugees are positioned as the biggest threats: “high population pressure in western Rwanda has led to the overutilization and degradation of the natural ecosystem” (UNDP, 2011: 11). “Whilst this area (i.e. the Gishwati ecosystem) was dominated by natural forests before the 1994 Genocide, the significant number of refugees returning from the DR Congo after the war has led to almost total denudation of the hilly landscape” (UNDP, 2011: 12). In turn, “rehabilitation of critical ecosystem services” is viewed as an essential fix for “buffering against climate change risks” (UNDP, 2011: 13). In 2011, thousands of households living in areas deemed the highest risk of flood were resettled through a 16 million USD project to sustainably intensify agricultural production by building terraces (Gebauer and Doevenspeck, 2015). The persistent emphasis on landscape engineering solutions (underlying which is a modernist vision of segregating nature/society and urban/rural) produced the peri-urban space of Mukamira (the primary site of 2007 landslides) in a way that made it vulnerable to floods. This illustrates how it is not only that the floods were produced by GBK landscape transformation (which was based on separating nature and agriculture) but also the peri-urban spaces that are adjacent to the forest were produced by this same logic, including political economies generated in GBK's socioecological fix (abundance of wage labor to establish pastures and forest plantations). The vulnerability of these communities to flood was thus produced through the socioecological fix and the way the subsequent refugee crisis mapped onto Gishwati's engineered landscape. Ironically, these floods—through the guise of climate resilience—have simultaneously emerged as the rationale for further landscape engineering and socioecological fix at the level of the Gishwati ecosystem, this time through the promotion of ecosystem services. This fluctuation of ideas about human–environment interaction and the way it channels capital illustrates the metabolism of conservation and development in Gishwati. The more things change, the more they remain the same.

A second lively element that emerged in Gishwati in the mid-2000s, and that has subsequently motivated the particularities of environmental governance in the region, was a small population of eastern chimpanzees (Pan troglodytes *schweinfurthii*). This population, estimated at 9 to 21 individuals was discovered in the fragment of primary forest in the Gishwati landscape (Barakabuye et al., 2007). Gishwati's isolated chimpanzees immediately drew the attention of the Great Ape Trust, an Iowa (US)-based non-profit sanctuary and research center founded by businessman Ted Townsend in 2002. Following meetings with Rwanda's ambassador to the US and other high-level officials, Townsend received government approval to reforest Gishwati and establish a Conservation Park (Tumwebaze, 2008). The Great Ape Trust obtained a Commitment to Action from the Clinton Global Initiative in 2007 in support of this project. Townsend's aims for reforestation coalesced in an ambitious plan to develop a “conservation corridor” to link the Gishwati chimpanzees with a larger population (around 800 individuals) in Nyungwe National Park, 50 kilometers to the south. The corridor plans were elaborate, for instance stipulating that only seeds collected from chimpanzee feces would be planted (Holden, 2008). Under the Gishwati Area Conservation Program (2008 to 2011) and then the Forest of Hope Association, these initiatives have led to a doubling of forest cover over a five-year period (Forest of Hope Association, 2012).

This group of chimpanzees came into existence—in the sense that they were discovered, researched, and used to mobilize millions of dollars in forest restoration—through the unique pattern of socionatural change in Gishwati. Through economic development, war, and political economies of refugee settlement, these chimpanzees have emerged as agents defining the region. Indeed, governance of Gishwati is increasingly being written in terms of the chimpanzee population's biological requirements. Work on Gishwati's chimpanzees has found that they are ecologically valuable, particularly in regenerating forest fragments due to their capacity for dispersing tree seeds (Chancellor et al., 2017). These scientific findings affirm the pre-existing commitment to constructing a forest corridor. Importantly, it is not the mere existence of the chimpanzees that appears to have stirred this enthusiasm, but specifically the presence of a breeding population in a highly fragmented area that is scientifically interesting (Chancellor et al., 2012). This suggests the role of scientists in reframing ways of seeing the forest. The plight of the chimpanzees also appears to have captivated global conservation interests. One tour company describes the Forest of Hope's mission as an effort “to keep the Gishwati Forest from becoming another casualty of the genocide” (http://kerdowney.com/2016/01/gishwati-forest/, accessed 8 March 2017). In re-casting the forest as important because of the chimpanzees, the Forest of Hope Association depicts the chimpanzees as castaways from political turmoil, refugees themselves, their isolation owing to regional instability and mismanagement by subsistence producers.

The plight of Gishwati’s chimpanzees and narrative of subsistence-led deforestation have also recently featured in the marketing of consumer products. For instance, London based Planet Organic promotes their ‘Gishwati Cloud Forest Climate Change Ground Coffee’ with the following description: “Gishwati use to host a big number different species of primates such as Chimpanzees and Golden monkeys. However, during the genocide, displaced refugees cleared it for subsistence farming. Deforestation was so intensive that by 2001 only 6km of native Gishwati forest remained. The coffee rewards 700 organic farmers for sustainable land management practices” (Planet Organic, 2019).

### Implementing a 'landscape approach' to conservation and development (2014–2019)

These visions of Gishwati—landscape of war and refugees, climate change-induced flooding, sanctuary for chimpanzees, and source of ecosystem services that can benefit downstream users—have converged since 2014 in the World Bank's Landscape Approach to Forest Restoration and Conservation (LAFREC) project. The main objective of this project (scheduled to run until December 2019) is to “demonstrate landscape management for enhanced environmental services and climate resilience in one priority landscape.” (World Bank, 2014: 4). Drawing on language of ecosystem services, LAFREC aims to make the Gishwati ecosystem a site of spatial fix. Curiously, this socioecological fix aims to respond to crises that were produced by the World Bank's previous projects and subsequent socionatural transformations. As the project proposal outlines, LAFREC emphasizes:

rehabilitating forests and biodiversity within the Gishwati and Mukura Forest Reserves, enhancing sustainable land management in the agricultural lands between them, and introducing silvo -pastoral approaches in the rangelands of the central former Gishwati Reserve. These interventions will be synergistic, enhancing biological connectivity at the landscape level in a fashion that offers strong potential for global recognition as a UNESCO Biosphere Reserve and longer-term re-orientation of the local economy towards nature-based tourism. (World Bank, 2014: 5)

The project has taken shape through Rwanda's broader Forest Landscape Restoration Program, an agenda of ecosystem-level governance that dovetails with the top-down landscape engineering for the sustainable intensification and commercialization of agricultural systems (Clay, 2018). The country's Land Use Master Plan structures this initiative, providing a “framework to enable decision-making from a landscape perspective – with a view to optimize a diversity of benefits (agricultural, infrastructure development, environmental etc)” (GoR, 2011). The Master Plan had already incorporated the Forest of Hope Association's plan for the Gishwati-Mukura corridor and the corridor plan has been adopted by the LAFREC project, a driving concern for which is enhancing biological connectivity for Gishwati's chimpanzees. This agenda will require resettling thousands of households to reforest the densely populated area between Gishwati and Mukura forests. To accomplish this, LAFREC's Resettlement Policy Framework has proposed “Significant investments in land use intensification would be offered to communities in return for restricting agriculture in the most vulnerable lands and establishing protection forests” (World Bank, 2014: 7). As Davis and Robbins (2018) argue of tree planting in India, the afforestation program in Gishwati functions as a form of biopolitics that extends state control over territory. By apportioning households land in newly constructed terraced areas where they can practice intensive agriculture, LAFREC also dovetails with the Government of Rwanda's countrywide Crop Intensification Program (CIP), a core policy mechanism to transition from subsistence to commercial agriculture (MINAGRI, 2011). The CIP has dramatically transformed Rwanda’s agrarian landscape and has exacerbated social inequalities in its drive to modernize agriculture (Ansoms et al., 2018; Clay, 2017). In this way, LAFREC appears to continue the dualism between human and nature, even though the project flies a banner of integrated landscape level management.

Still more paradoxically, LAFREC appears to aim to return the landscape to the state it was in 40 years ago—before large-scale landscape manipulation for dairy and forestry industrialization. Indeed, the intended outcomes of biodiversity and nature-based tourism are essentially opposites of the GBK project's intent to develop of natural resource-based industry. And a synergistic management plan premised on integrating multiple uses (forestry, pastoralism, agriculture, and tourism) stands in stark contrast to the modernization-based strategy that purposively separated these uses. While the logic of the GBK projects was that forest conservation depended on removing cattle from the forest, the current silvo-pastoral plan is calling for putting them right back in. Yet, getting back to that state of silvo-pastoral synergy requires substantial alteration of Gishwati's socionature, a socioecological fix that purports to resolve a new set of crises. In this sense, LAFREC is premised on the same modernizing motif as GBK. The protocols of both projects rely on (1) categorizing the area as degraded and in need of coordinated governance and (2) delimiting interventions with respect to spatial and quantifiable attributes. With GBK, success was pinned on area of converted pasture and number of cows. With LAFREC, it is biodiversity, which in turn is defined by the extent of land reforested and where the trees are positioned so as to construct a corridor. By hinging on spatial-economic measures of success, these governance initiatives have, in similar ways, avoided recognition of the very socioecological processes that supposedly drive the projects. LAFREC arguably only appears to differ from GBK because it is being articulated in relation to the material results of GBK (floods and chimpanzees) and the marginalized groups blamed for environmental degradation (refugees and subsistence farmers).

## Towards a landscape of inclusive hope

Despite the different strategies and objectives of the GBK and LAFREC projects, they are united in the glaring logic of landscape engineering as a means to achieve conservation and development goals. Analyzing trajectories of change and continuity through this case study illustrates how power relations and social inequality are embedded in the metabolism of social-environmental crises and their fixes. Sinking capital into the Gishwati landscape repeatedly emerges as a solution to socioecological crisis while, at the same time, these socioecological fixes enable trajectories of social–environmental change that continue to generate social inequalities and environmental destruction: in other words subsequent crises.

In documenting this metabolic process, this narrative highlights how the shift to ecosystem-level governance and reforestation may be an effort to reconstruct the legitimacy of development organizations as pseudo-conservation institutions. As Ekers (2015) suggests about government investments in industrial forestry in 1930s Canada, crises of legitimacy can be key rationales for implementing socioecological fixes. With this analysis of the World Bank's transitions over a 40-year period, this paper engages with the idea that sustainable development is a fix to maintain trajectories of economic growth in the face of intersecting global economic and ecological crises (Temenos and McCann, 2012). It considers how re-scaling to the level of an ecosystem (Cohen and McCarthy, 2015) is a political ecological endeavor that can be entangled with development and conservation, the biophysical environment, and social inequalities. That the ecosystem has emerged as the unit of governance for these complex processes illustrates the degree to which such logics are deeply entangled in the ‘capitalocene’ (Moore, 2011, 2017). Along these lines, this paper has discussed how socionatural hybrids emerge and are constantly reworked through historical geographical processes (Swyngedouw, 1999, 2003) of conservation and development. Similar to Zalik's (2015) depiction of oil drilling frontiers, I have argued in this paper that governance schemes in Gishwati serve to enroll and celebrate ecosystems into capitalist production while also quieting socioecological controversies by hiding them within broader institutional processes and defined spatial arenas. In the case of LAFREC, the process is reforestation and the spatial unit is the ecosystem.

It has not been my intention to depict landscape/ecosystem approaches as blind or superficial. That would overlook the more important point, which is that these governance schemes can be driven by visions of a particular—and contextually specific—socionature. In Gishwati, these imaginaries are continually reframed with reference to antecedent and cotemporaneous projects, events, and narratives that are locally relevant. As Prudham (2003) argues, attempts at deriving economic value in the production of nature are not static but rather “unfold, changing in response to new opportunities and constraints emerging from a dialectical conversation between social and environmental change” (p. 638, citing Harvey, 1996). It is essential to consider how processes of development and conservation intersect over time in the uneven production of nature. In the case of Gishwati, the history of large-scale land-management begets more large-scale management. The rapid transformations of the Gishwati landscape and associated effects (major forest clearing, floods, isolated chimpanzees) have made Gishwati a laboratory of socioecological fixes.

The current articulation of ecosystem-level conservation in Gishwati is a chimeric incarnation of attempts to bend environment–society processes to the allegedly natural logics of capitalism and ecology. These logics have continually churned out injustice. Gishwati was given the Forest of Hope moniker due to the small chimpanzee population's miraculous survival. In an effort to garner capital for the reforestation effort, the chimpanzees were depicted as refugees themselves. At the same time, human refugees in Gishwati were made the scapegoats of forest destruction, regardless of the fact that much of the deforestation was caused by a World Bank project in the 1980s and by commercial tea production in the 1960s–1970s.

Given the persistent marginalization of local concerns, it is essential to ask how LAFREC and other landscape/ecosystem approaches to conservation might cultivate a more inclusive vision of hope. A core objective of LAFREC is to submit a proposal to make Gishwati a UNESCO Biosphere Reserve, a prospect that could further entrench these patterns of inequality if care is not taken to recognize local voices and diverse knowledges about the landscape. Fixes that inject capital and technology into an uneven landscape will likely continue to recreate injustice unless the deep roots of inequality and power imbalance are addressed.
